# Online Frequency Response Analysis of Electric Machinery through an Active Coupling System Based on Power Electronics

**DOI:** 10.3390/s21238057

**Published:** 2021-12-02

**Authors:** Wilson Cesar Sant’Ana, Germano Lambert-Torres, Erik Leandro Bonaldi, Bruno Reno Gama, Tiago Goncalves Zacarias, Isac Antonio dos Santos Areias, Daniel de Almeida Arantes, Frederico de Oliveira Assuncao, Mateus Mendes Campos, Fabio Monteiro Steiner

**Affiliations:** 1 Gnarus Institute, Itajuba 37500-052, MG, Brazil; germano@institutognarus.com.br (G.L.-T.); erik@institutognarus.com.br (E.L.B.); tiago@institutognarus.com.br (T.G.Z.); fredeoa@gmail.com (F.d.O.A.); mateusmcampos@unifei.edu.br (M.M.C.); 2 Pro-Reitoria de Pesquisa e Pos-Graduacao (PRPPG), Itajuba Federal University, Itajuba 37500-903, MG, Brazil; brrgama@gmail.com (B.R.G.); isacareias@gmail.com (I.A.d.S.A.); daniel_arantes@unifei.edu.br (D.d.A.A.); 3 EDF Norte Fluminense, Macae 27910-970, RJ, Brazil; fabio.steiner@edfnf.com.br

**Keywords:** condition based monitoring, failure diagnosis, electric machinery, frequency response analysis, modular multilevel converters, power electronics, predictive maintenance

## Abstract

This paper presents an innovative concept for the online application of Frequency Response Analysis (FRA). FRA is a well known technique that is applied to detect damage in electric machinery. As an offline technique, the machine under testing has to be removed from service—which may cause loss of production. Experimental adaptations of FRA to online operation are usually based on the use of passive high pass coupling—which, ideally, should provide attenuation to the grid voltage, and at the same time, allow the high frequency FRA signals to be injected at the machine. In practice, however, the passive coupling results in a trade-off between the required attenuation and the useful area obtained at the FRA spectra. This paper proposes the use of an active coupling system, based on power electronics, in order to cancel the grid voltage at the terminals of FRA equipment and allow its safe connection to an energized machine. The paper presents the basic concepts of FRA and the issue of online measurements. It also presents basic concepts about power electronics converters and the operating principles of the Modular Multilevel Converter, which enables the generation of an output voltage with low THD, which is important for tracking the grid voltage with minimum error.

## 1. Introduction

Condition Based Maintenance (CBM) is the continuous monitoring and analysis of some parameters of an asset in order to evaluate some tendency toward a decrease in performance or incipient fault [1]. According to Kafeel et al. [2], the parameters most often monitored are current, sound and vibration. Ribeiro et al. [3] presented a monitoring system for the currents of hydro-generators and applied a Fast Fourier Transform (FFT) over the Park transform of the currents. Variants of Fourier transforms are usually used for vibration and acoustic signals, as presented in [2]. Verellen et al. [4] presented a non-invasive monitoring system for acoustic signals using a sparse microphone array in order to detect bearing failures in rotating machines. Conventional vibration analysis use accelerometers, which are contact sensors and need to be attached to the part under investigation [4]. An interesting approach to contactless monitoring of vibration was proposed by Śmieja et al. [5], where image processing is employed.

Another technique, Frequency Response Analysis (FRA), has been widely used for the offline diagnosis of transformers, in which case its application is guided by the standard IEEE-Std-C57.149 [6], and several pieces of commercial equipment are available on the market for this purpose. According to Blanquez et al. [7], despite the great developments in FRA for transformers, its application for rotating machines (motors and generators) is still under research. Additionally, some recent interesting applications of FRA include the work of Guerrero et al. [8] on the detection of fluid degradation, the work of Al-Ameri et al. [9] on the detection of failures in transformer tap changers and the work of Kumar et al. [10] on the identification of the location of an inter-turn insulation fault in a transformer winding.

The FRA technique is based on the comparison of spectra, either of admittance/impedance or of any relation of gains in the tested machine. Usually, a reference spectrum, known as baseline or fingerprint, is measured with the machine in a healthy condition, and this spectrum is compared against new spectra measured during the machine’s lifespan. If there is a difference in the comparison, this is an indication that the intrinsic parameters of inductances, capacitances and resistances have changed and might indicate the beginning of a failure.

According to Pramanik et al. [11], the baseline or reference signature is not always available, specially for old machines. Hence, they proposed the injection of two equal and opposite polarity sinusoidal excitation from the two ends of a winding. For a healthy machine, the two measured admittances/impedances are likely to be identical. Conversely, a discrepancy between the two measurements is an indication of failure. Some other alternatives to the need for historical data, as suggested in the standard IEEE-Std-C57.149 [6], may be the use as reference the measurements performed on a “sister machine”, or even, in another phase of the same machine —although some concerns have been raised for both cases. Additionally, it is common sense in CBM to always consider a trend curve of the measurements. Hence, even if the measurements have been started at a later point in the lifetime of the machine, the analysis can start from that point on. The trend based approach, also, adds more tolerance to imprecision in the measurements and is useful when uncontrolled external factors are an inevitable part of the process. These uncontrollable factors can be related to temperature and humidity, which are known to affect the measurements [12,13]. Additionally, in a rotating machine, the angular position of the rotor produces variations in the inductive regions of spectra due to differences in the machine’s air gap [14,15].

According to Ryder [16], the comparisons performed between the measured spectra are usually visual and require an experienced observer in order to identify changes in the shapes of the curves and in the resonant frequencies. Additionally, according to Ryder [16], the main problem with this method is that the expert’s opinion may lack objectivity. Hence, it is proposed the use of statistical indexes in order to objectively indicate the amount of agreement/disagreement between the spectra. A literature survey on these indexes was written by us [17]. However, according to Al-Ameri et al. [18], consistent interpretations of FRA signatures are still challenging due to the lack of widely accepted FRA codes. Recent efforts in order to improve the interpretation of FRA signatures have been reported, usually using some form of machine learning, such as the works of Ferreira et al. [19] and Li et al. [20].

Another aspect of great interest is the use of FRA on an energized machine—without the need to stop and disconnect the machine in order for the tests to be performed, hence, without loss of production—in what is called online operation. Gomez-Luna et al. [21] presented a review on the efforts towards online applications of FRA. These methods can be based on the measurement of spectra caused by either an impulsive nonperiodic excitation (named IFRA, or Impulse Frequency Response Analysis) or by a sweep in frequency of a periodic sinusoidal excitation (named SFRA, or Sweep Frequency Response Analysis).

In the case of IFRA, it is assumed that the excitation impulse will have enough frequency components in order to extract the resulting spectra using some domain transformation tool—conventionally, the FFT. As the excitation impulse is not periodic, and periodicity is a requirement for application of FFT, recent works in the literature, such as Zhao et al. [22], explored other transformation tools more appropriate for non periodic signals, such as wavelets transforms. The excitation impulses can be either generated in a controlled way (using an impulse generator) or an uncontrolled way (using disturbances that naturally occur in the system, such as the opening and closing of a breaker or atmospheric events [21]). According to [21], controlled excitation has the advantage of a better bandwidth for the spectra—although the measurement of uncontrolled transients is less intrusive and requires less complex instrumentation. Furthermore, concerning the use of controlled excitation on IFRA, the recent works of Arunachalam et al. [23,24] show that, whenever the exciting impulse has a moderate high voltage, even partial discharges can be measured—in addition to FRA.

In the case of SFRA, low voltage sinusoidal excitation is applied in a controlled and precise frequency sweep (from hertz to megahertz). According to Ryder [16], the main advantages of the SFRA in relation to IFRA are the better signal to noise ratio and nearly equal accuracy across the whole measurement range. Furthermore, the work of Rahimpour et al. [25] adds that, with SFRA, there is a reduced need for complex signal processing—as no domain transformations, such as FFT or wavelets, are required. However, for online applications, the low voltage signal generator responsible for the frequency sweep has to be connected to the energized machine through some sort of coupling device; otherwise, the low voltage equipment would be damaged due to the operating voltage of the energized machine (at hundreds or, even, thousands of Volts).

Besides the attenuation of the operating voltage, the coupling device should not interfere with the high frequency signals being injected into the energized machine. The review presented by Gomez-Luna et al. [21] also highlights that these requirements are very similar to the ones required in Power-Line Communication (PLC), where the high frequency modulated data are injected into an energized power line through passive high-pass filters. For power transformers, usually there are bushing taps (which act as high-pass capacitive voltage dividers) that are used experimentally for online FRA. For rotating machines (motors and generators) and transformers without bushing taps, following the PLC approaches, passive high-pass filters can be used.

We studied in [26] the effects of a passive high-pass filter over the measured FRA spectrum. An ideal high-pass filter must offer high impedance at low frequencies and low impedance at high frequencies. It has been found, however, that, in order to achieve proper attenuation of the grid voltage, the impedance of a real passive filter must still be high enough to have an impact on the measurements—producing a “shadow” over the low frequency region of the measured spectrum. The same phenomenon has also been noted by Bagheri et al. [27] while studying bushing taps.

This paper proposes a novel concept of active coupling of a FRA low voltage equipment to energized machinery (generator, motor or transformer). The proposed active coupling device is based on the active power filtering concept, widely used in power electronics to compensate harmonics. Here, however, the active coupling generates the exact opposite voltage of the grid (in order to cancel it), so that the FRA equipment is still connected to the energized machine but at a minimum resulting residual voltage. Although the application in this paper of the active coupling is specifically online FRA, it can also be used for connection of PLC modems or any low voltage equipment that one desires to be connected to an energized grid.

The rest of the paper is organized as follows: Section 2 presents the fundamental concepts of the proposition—which have been divided into subsections. Section 2.1 presents the basic concepts about FRA, including three of the most used statistical indexes. Section 2.2 presents an explanation on why passive filters, and also capacitive bushing taps, create a “shadow” over the low frequency regions of the FRA spectra. Section 2.3 presents the main idea of the active coupling based on a power electronics converter. Although this system could be implemented using any of the main topologies for DC–AC converters, a multilevel topology is presented—as it is modular and can be easily adapted to higher voltage levels. Furthermore, basic concepts about power electronics converters are presented in this subsection. Section 3 presents the experimental results. Finally, Section 4 presents the main conclusions of the work and some opportunities for future research.

## 2. Methods

### 2.1. Frequency Response Analysis

According to [28], FRA can be basically described as the comparison of two spectra, taken at different instants of time of the machine’s lifespan. Differences between a given spectrum and its baseline spectrum may indicate the onset of a failure.

The literature presents different types of spectra to be compared: impedance/admittance spectra and the spectra of any relation between two measurable quantities of the machine. Henceforth, this work focuses on the impedance spectra of the machine under testing.

**Figure 1:**
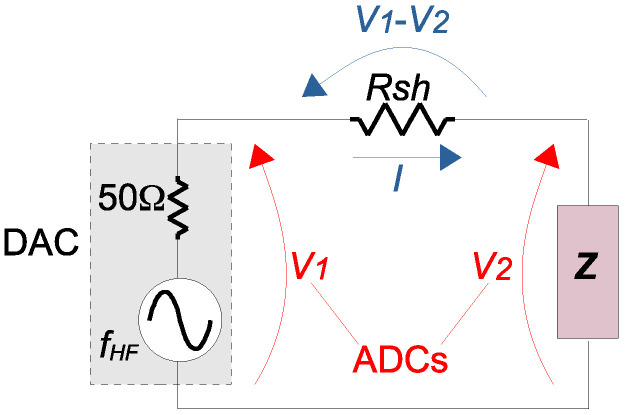
Measurement circuit for frequency sweep of the impedance under test *Z*, using general purpose ADCs and DACs and a shunt resistor $R_{sh}$—Redraw based on [15].

Figure 1 presents the measurement circuit used to obtain the spectra. It is composed of a programmable analog output (which usually has an output impedance of 50 Ω) and two general purpose analog inputs (which usually have input impedances of 1 MΩ). It is important to note that this circuit can be implemented with general purpose ADCs and DACs and is different from the commercial FRA equipment (whose analog inputs also have 50 Ω impedance and are connect to the circuit to be measured differently), as presented in [15,29]. Considering the circuit of Figure 1, the current flowing through the impedance to be tested is indirectly obtained using a shunt resistor $R_{sh}$. Based on the amplitude of the signal $V_{2}$ ($\left|{\vec{V}}_{2}\right|$) and on the amplitude of the signal resulting from the subtraction of $V_{2}$ from $V_{1}$ ($\left|{\vec{V}}_{1}-{\vec{V}}_{2}\right|$), the impedance *Z* can be determined as Equation (1). The Appendix A presents a method to extract the amplitude of a signal through DFT.

(1) $$\left|\vec{Z}\right|=\frac{\left|{\vec{V}}_{2}\right|}{\left|\vec{I}\right|}=\frac{\left|{\vec{V}}_{2}\right|}{\left|\frac{{\vec{V}}_{1}-{\vec{V}}_{2}}{R_{sh}}\right|}=R_{sh}\cdot \frac{\left|{\vec{V}}_{2}\right|}{\left|{\vec{V}}_{1}-{\vec{V}}_{2}\right|},$$

where $R_{sh}$ is the shunt resistor used to indirectly obtain the current *I* from the two measured analog voltages $V_{1}$ and $V_{2}$.

It is important to note that Equation (1) calculates only a point of impedance—specifically at the frequency being injected ($f_{HF}$ in Figure 1). With the variation of $f_{HF}$ within a large range of frequencies, an impedance spectrum is obtained. Then this obtained spectrum is compared against its historical data, in order to detect discrepancies.

As the FRA technique is based on the comparison of spectra obtained at different stages of the machine lifespan and as these comparisons may be subjective to the experience of the analyst [17], statistical indexes have been proposed in order to achieve more objective diagnoses. A survey on these indexes is presented in [17]. The advantage of using such indexes is that the result of each comparison is a numerical score that indicates, quantitatively, how much a given spectrum is differing from its baseline condition. However, each of these indexes has its own particularities and respond differently to different types of damages. Due to these different responses, [30] recommends the use of more than one index, in a complimentary way—in order to avoid underestimation or exaggeration of certain deviations. Equations (2)–(4) present three of the most used indexes. These same indexes were used in the analysis of the experimental results presented in Section 3. For these three indexes, two spectra of *n* frequencies are considered (as $X=\{x_{1},x_{2},\ldots ,x_{n}\}$ and $Y=\{y_{1},y_{2},\ldots ,y_{n}\}$), where the *X* spectrum represents the baseline spectrum.

The Absolute Sum of Logarithmic Error (ASLE) index, as discussed by Kim et al. in [31], is defined as Equation (2).

(2) $$ASLE=\frac{\sum_{i=1}^{n}\left|20log_{10}y_{i}-20log_{10}x_{i}\right|}{n}.$$

The Sum Squared Max–Min ratio error (SSMMRE) index, as discussed by Kim et al. in [31], is defined as Equation (3).

(3) $$SSMMRE=\frac{\sum_{i=1}^{n}{\left(\frac{max(x_{i},y_{i})}{min(x_{i},y_{i})}-1\right)}^{2}}{n}.$$

The Minimum–Maximum ratio (MM) index, as discussed by Secue and Mombello in [32], is defined as Equation (4).

(4) $$MM=\frac{\sum_{i=1}^{n}min\left(y_{i},x_{i}\right)}{\sum_{i=1}^{n}max\left(y_{i},x_{i}\right)}.$$

Furthermore, according to [26], the FRA is, essentially, an offline method. This implies that the machine being tested must be disconnected from the grid in order for the tests to be performed—otherwise, the grid voltage may damage the low voltage electronics of the FRA equipment. In order to perform online FRA measurements to an energized machine, a coupling system is required. Section 2.2 presents the issues concerning traditional passive coupling. Section 2.3 presents the proposed solution, which is the use of a power electronics based active coupling.

### 2.2. Passive Coupling and Its Effects on the Spectra

**Figure 2:**
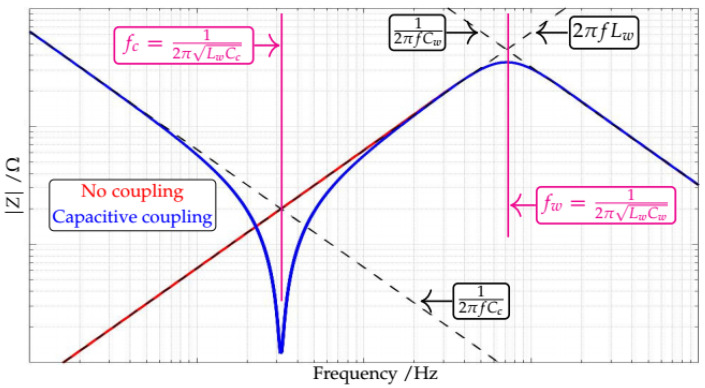
Coupling “shadow” over measured impedance spectrum—Redraw based on [26].

Figure 2 presents the effect of a passive capacitive coupling on the measurement of a fictitious motor stator winding impedance spectrum. For the motor winding we consider a parallel association of the stator inductance ($L_{w}$) with a parasitic capacitance ($C_{w}$, which predominates at higher frequencies). The red plot in the figure represents the impedance spectrum measured without any coupling. At this condition, at low frequencies (before the resonance between $L_{w}$ and $C_{w}$, at $f_{w}=\frac{1}{2\pi \sqrt{L_{w}C_{w}}}$), the inductive part predominates at the measurements—hence, the red plot follows the ascending dashed asymptote given by $2\pi fL_{w}$. Furthermore, at high frequencies (after the resonance at $f_{w}$), the capacitive part predominates at the measurements—hence, the red plot follows the descending dashed asymptote given by $\frac{1}{2\pi fC_{w}}$.

The blue plot in Figure 2 represents the impedance spectrum measured with a passive capacitive coupling. At this condition, at low frequencies (before the resonance between the coupling capacitor $C_{c}$ and the winding inductance $L_{w}$, at $f_{c}=\frac{1}{2\pi \sqrt{L_{w}C_{c}}}$), the coupling capacitor predominates at the measurements—hence, the blue plot follows the descending dashed asymptote given by $\frac{1}{2\pi fC_{c}}$. At middle frequencies (higher than $f_{c}$ and lower than $f_{w}$), the winding inductance $L_{w}$ predominates—hence, the blue plot follows the ascending dashed asymptote given by $2\pi fL_{w}$. At high frequencies (after the resonance at $f_{w}$), the winding capacitance $C_{w}$ predominates—hence, the blue plot follows the descending dashed asymptote given by $\frac{1}{2\pi fC_{w}}$.

From Figure 2, it can be noted that the passive coupling has produced a “shadow” over the low frequency region (below the resonance $f_{c}$, between the capacitance of the coupling $C_{c}$ and the winding inductance $L_{w}$) of the spectrum. Hence, while using a passive coupling, there will be a trade off between the desired attenuation at the grid voltage and the useful “not shadowed” region of the measured spectrum [26].

### 2.3. Proposed Active Coupling Based on Power Electronics Converter

**Figure 3:**
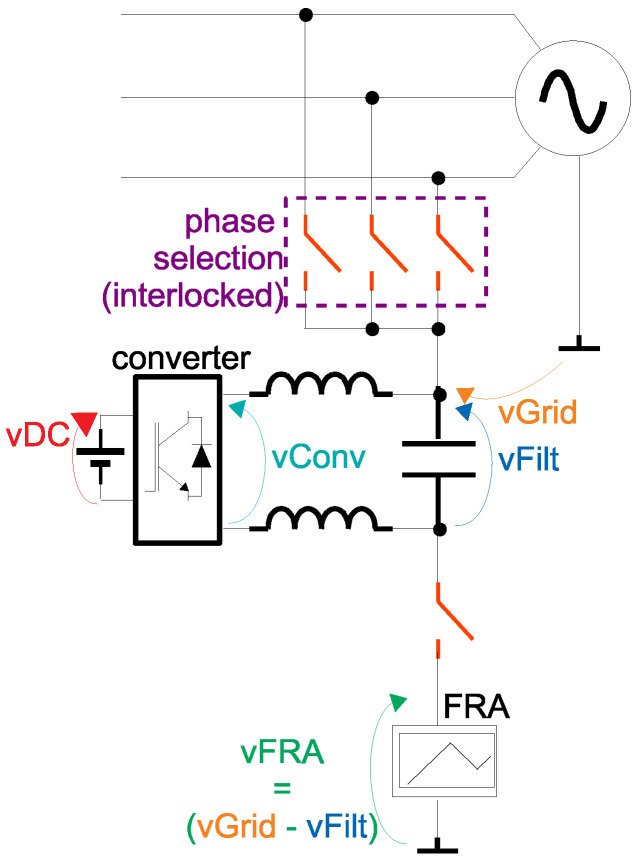
Proposed active voltage block for safe online operation of the FRA or any electronic system operating at a higher frequency than the grid.

In order to be able to couple an electronic measurement equipment to an energized winding of an electric machinery (generator, motor or transformer), this paper proposes an innovative concept of active coupling based on power electronics. The operating principle of the active coupling is similar to the principle of a series active power filter (widely used in power systems in order to compensate for voltage harmonics and voltage sags [33]). Figure 3 presents the proposed active voltage blocker, with indication of the main voltages that are going to be used in the analysis.

The orange arrow in Figure 3 represents the grid phase voltage (line-to-ground) vGrid which we desire to eliminate from the terminals of the FRA electronic measurement system (indicated by the green arrow vFRA). It is known, from Kirchhoff’s voltage law, that the sum of the voltages inside a loop must be zero. Hence, if a power electronics converter is inserted in series between the grid and the FRA, generating a filtered voltage indicated by the blue arrow vFilt, the voltage of the grid can be completely eliminated at the FRA terminals if vFilt is controlled to be exactly the same as vGrid.

The power electronics DC–AC converter, however, can only chop its DC input voltage (represented by the red arrow vDC in Figure 3) proportionally to its reference command and re-arrange the chopped voltages into an AC staircase pattern (indicated by the cyan arrow vConv). In order to extract the average information (i.e., the filtered signal vFilt) contained in the unfiltered output of converter vConv, a passive LC filter is usually used. As the LC filter can produce a phase delay and amplitude attenuation on the filtered signal, a closed loop controller is also required—in order to produce a vFilt voltage as close as possible to vGrid.

Particularly in the experimental results of Section 3, the topology of the power electronics DC–AC converter is a Modular Multilevel Converter (MMC)—although other known topologies could have been used. The advantage of the chosen topology is the smaller THD at vConv—which requires a smaller passive filter at its output in order to obtain vFilt. Furthermore, as a multilevel topology, the voltage drop at each IGBT is divided among the submodules—which makes this topology ideal for operation on higher voltages. Section 2.3.1 describes the MMC topology. An important aspect to be noted in this application is that the power electronics converter does not require high current rating (as it is connected in series only with the FRA equipment and there will be no current at fundamental frequency flowing through them)—however, the IGBTs of the converter must be rated according to the voltage share at each level.

Furthermore, particularly in the experimental results of Section 3, the type of controller used to make vFilt track vGrid is the Proportional Plus Resonant (PR) controller—although other known controller structures could have been used. The advantage of this type of controller is that it enables tracking of sinusoidal references (without steady state errors) without the need of coordinate transformations. Section 2.3.2 describes the PR controller.

#### 2.3.1. Modular Multilevel Converter—MMC

The work of Debnath et al. [34] presents a review on aspects related to the MMC converter. Among the advantages, we can highlight the modularity and scalability in order to meet any voltage level requirement and its superior harmonic performance—as, with the increase in the number of voltage levels, the resulting staircase pattern will be closer to sinusoidal, even without filtering.

**Figure 4:**
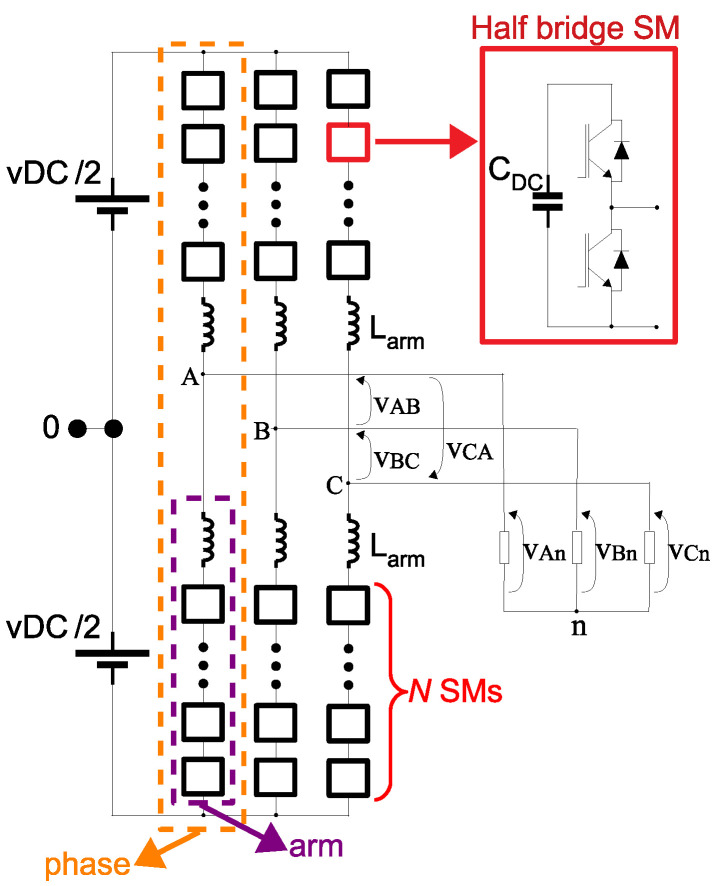
General schematics of a three-phase MMC (2 arms/phase and N SMs/arm).

Figure 4 presents the general schematics of a three-phase MMC converter. Each phase of the converter is composed by two arms (a lower arm and an upper arm). Each arm is composed by *N* submodules (SMs) and an arm inductance. The function of the arm inductance is to suppress the high frequency components in the arm current [34]. The work of Debnath et al. [34] lists several of the circuits that can be used as SMs in the MMC. For this work, the half-bridge SM was chosen, as it is the one that uses the least components and is the simplest to control.

**Figure 5:**
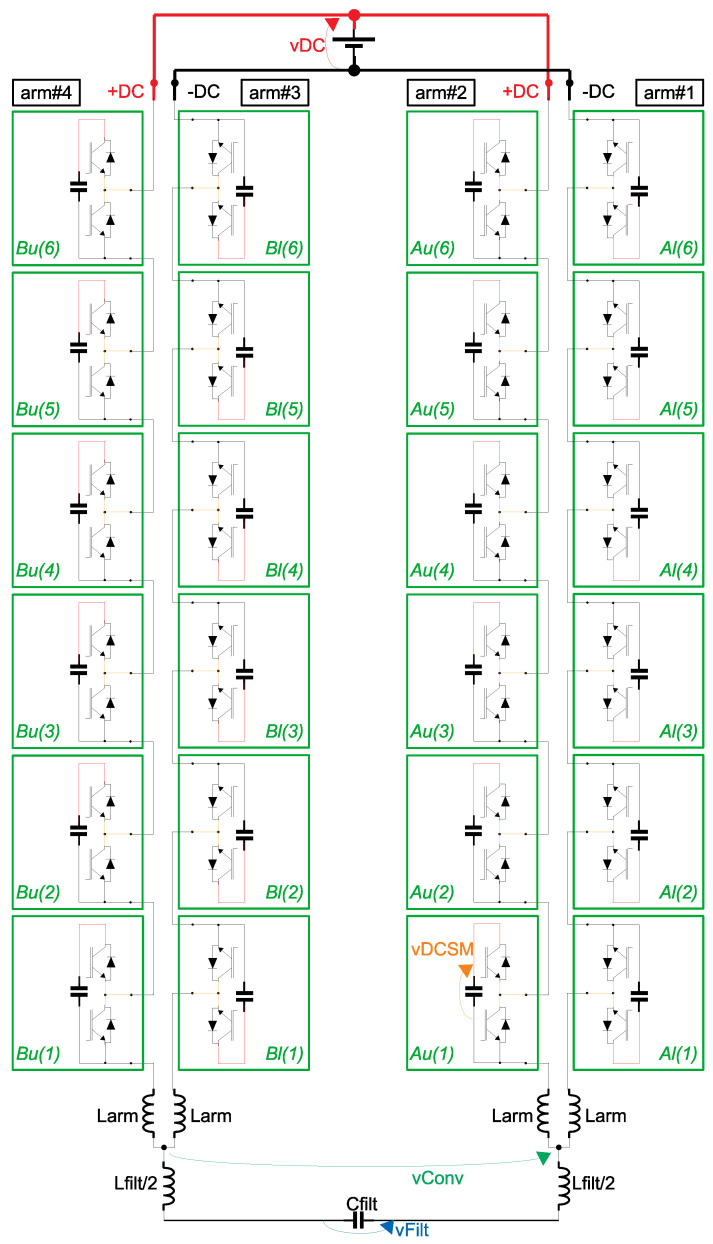
Schematics of the Modular Multilevel Converter (MMC) topology (bi-phase, 4 arms, N=6 SMs/arm).

As presented in Figure 3, in case of the application as active voltage blocker for the FRA equipment, a single-phase converter is connected between one the phases and the grounded FRA. For single-phase applications, only one of the phases of the MMC of Figure 4 could be used, as long as the load is connected to the middle point of the DC source (indicated by the point 0 in Figure 4). This type of connection, however, is sensitive to unbalance in the DC voltage in the upper and lower parts of the figure. Hence, for the active voltage blocker, as a more precise voltage vConv is required, two phases of the MMC of Figure 4 are used—which eliminates the necessity for connection to the middle point of the DC source. Figure 5 presents a bi-phase MMC, with 6 SMs/arm (the same configuration which is going to be used in the experiments of Section 3).

The SMs in Figure 5 are indicated by their phase (either *A* or *B*), their arm position (either *l* for lower arm or *u* for upper arm) and their SM number within each arm (1 to 6). In order to save space in the figure, the positioning of the upper and lower arms are folded around the arm inductances—which implies that all SMs in the lower arms (Al(1…6) and Bl(1…6)) are drawn upside down in relation to the SMs in the upper arms (Au(1…6) and Bu(1…6)).

**Figure 6:**
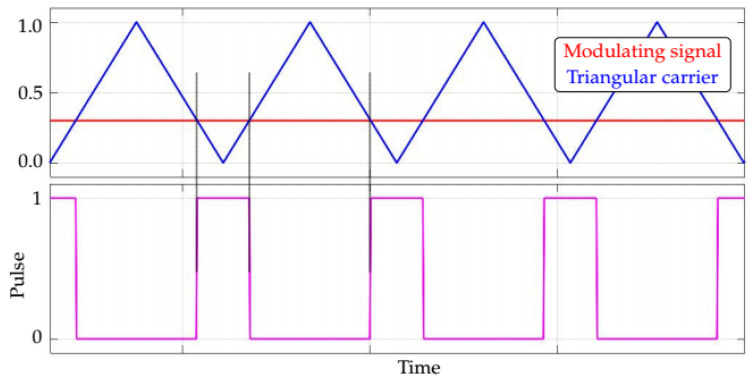
Basic PWM pulse generation.

In order to make the IGBTs of the converter switch proportionally to the desired voltage at the output of the converter, a PWM technique is used. Figure 6 presents the working principle of the basic building block of the majority of the PWM techniques, based on the comparison of the reference signal (also known as modulating signal) with a triangular carrier. Whenever the modulating signal has a value greater than (or equals) the value of the triangular carrier, the command pulse to the IGBTs of a particular SM is activated (logic level “1”). Conversely, whenever the modulating signal has a value less than the value of the triangular carrier, the command pulse to the IGBTs of that particular SM is deactivated (logic level “0”).

For each phase of the converter, the modulating signal of the upper arm must have a 180${}^{\circ }$ phase difference in relation to the respective lower arm. Furthermore, there must be a phase difference between each phase of the converter. For a three-phase MMC (Figure 4), the phase difference of the modulating signals (from one phase to another) should be 120${}^{\circ }$. However, for a bi-phase MMC, a 180${}^{\circ }$ phase difference is used.

Each SM of the MMC will have its own triangular carrier. The literature presents many different techniques for PWM in multilevel converters—where the differences are mainly related to the shifts of the triangular carrier of a SM in relation to the others. These shifts may be in relation to amplitude of the modulating signal (level shift PWM) or in relation to phase angle among the carriers (phase shift PWM). In this work, the phase shift technique is preferred, as the semiconductor stresses and the power handled by each SM are evenly distributed [35].

The work of Li et al. [35] presents an analysis of the phase shifts between each triangular carrier and the way they affect either the harmonic content of the output voltage or the arm current. In this current work, it is more important to minimize the harmonic content of the output voltage (as it is desired to have vFilt as close as possible to vGrid in Figure 3). The phase shifts between the triangular carriers of the lower arms ($\theta_{l}\left(i\right)$) can be calculated as Equation (5).

(5) $$\theta_{l}\left(i\right)=\frac{360^{\circ }}{N}\cdot \left(i-1\right),$$

where *N* is the number of SMs per arm and *i* an index that varies from 1 to *N*. With respect to Figure 5 (where N=6), $\theta_{l}\left(i\right)$ can be either $A_{l}\left(i=1\ldots 6\right)$ or $B_{l}\left(i=1\ldots 6\right)$.

The phase shifts between the triangular carriers of the upper arms ($\theta_{u}\left(i\right)$) can be calculated based on a constant phase difference θ between $\theta_{l}\left(i\right)$ and $\theta_{u}\left(i\right)$, as Equation (6). When it is desired to minimize the harmonic content at the output voltage, the constant angle θ can be calculated as Equation (7), according to [35].

(6) $$\theta_{u}\left(i\right)=\theta +\theta_{l}\left(i\right).$$

(7) $$\theta =\left\{\begin{array}{c} 0^{\circ },\;\;\;\;\;\;\;\;\;\;\;\;\;\;\;\mathrm{when}N\mathrm{is}\mathrm{odd};\\ 180^{\circ }/N,\;\;\;\;\;\;\;\;\mathrm{when}N\mathrm{is}\mathrm{even}. \end{array}\right.$$

With respect to Figure 5 (where N=6—hence, an even number), $\theta =30^{\circ }$. Table 1 summarizes the PWM parameters for this particular case. The parameter mod refers to the phase angle between the modulating signals.

#### 2.3.2. Proportional plus Resonant Controller—PR

In order to well remove the grid voltage on the FRA (or any other low voltage electronic equipment to be connected to the grid, such as PLC modems) of Figure 3, the filtered voltage generated by the converter vFilt must track as close as possible the grid voltage vGrid (i.e., vFilt≈vGrid)—hence, a closed loop controller must be used.

For three-phase systems, usually, the control loops are performed on d-q reference frames; thus, the control variables are constant in relation to time. Hence, PI controllers can be used without major issues [36]. However, in the case of single-phase systems, the control variables have sinusoidal references, which cannot be properly tracked by regular PI controllers without steady state errors [36].

Considering the application of the active voltage blocker, any steady state error may imply in a voltage large enough to damage the FRA system. Hence, in order to properly track sinusoidal references, a Proportional plus Resonant Controller (PR) is more appropriate.

**Figure 7:**
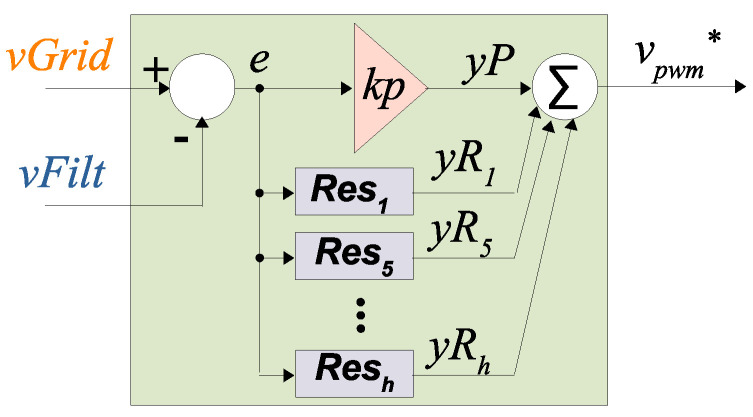
Proportional plus Resonant Controller—Redraw based on [37].

Figure 7 presents the PR controller. At each sampling instant, the measured vFilt is compared against its reference value (vGrid), generating an error signal *e*. The PR controller will act on the error *e* by means of a proportional controller $k_{p}$ (in order to improve transient response) and several resonant controllers ($Res_{1\ldots h}$, in order to minimize steady state errors at each of the resonant frequencies, or harmonics) [37].

The transfer function at the continuous frequency domain *s* for each of the resonant controllers of Figure 7 is given by Equation (8).

(8) $$Res_{h}\left(s\right)=\frac{yR_{h}\left(s\right)}{e(s)}=\frac{k_{Rh}\cdot s}{s^{2}+\omega_{h}^{2}},$$

where $k_{Rh}$ is the resonant gain at the harmonic frequency $\omega_{h}$.

In order to implement the controller in a digital signal processor, Equation (8) must be discretized. Using the well known trapezoidal/Tustin method [38], the difference Equation (9) is obtained.

(9) $${v_{pwm}}^{*}\left(t\right)=k_{P}\cdot e\left(t\right)+\sum_{h}b_{h}\cdot k_{Rh}\cdot \left[e(t)-e(t-2)\right]-\sum_{h}\left[a_{1h}\cdot y_{Rh}\left(t-1\right)+a_{2h}\cdot y_{Rh}\left(t-2\right)\right],$$

where $a_{1h}$, $a_{2h}$ and $b_{h}$ are parameters calculated by Equation (10), which are dependent on the sampling time $T_{s}=1/f_{s}$ and on the harmonic frequencies $\omega_{h}$ [36,37].

(10) $$\left\{\begin{array}{c} a_{0h}=4/T_{s}^{2}+\omega_{h}^{2};\\ a_{1h}=\left[-8/T_{s}^{2}+2\cdot \omega_{h}^{2}\right]/a_{0h};\\ a_{2h}=1;\\ b_{h}=\left[2/T_{s}\right]/a_{0h}. \end{array}\right.$$

The output ${v_{pwm}}^{*}$ is the reference (or modulating) signal of the multilevel PWM modulator (discussed in Section 2.3.1).

## 3. Results and Discussion

**Figure 8:**
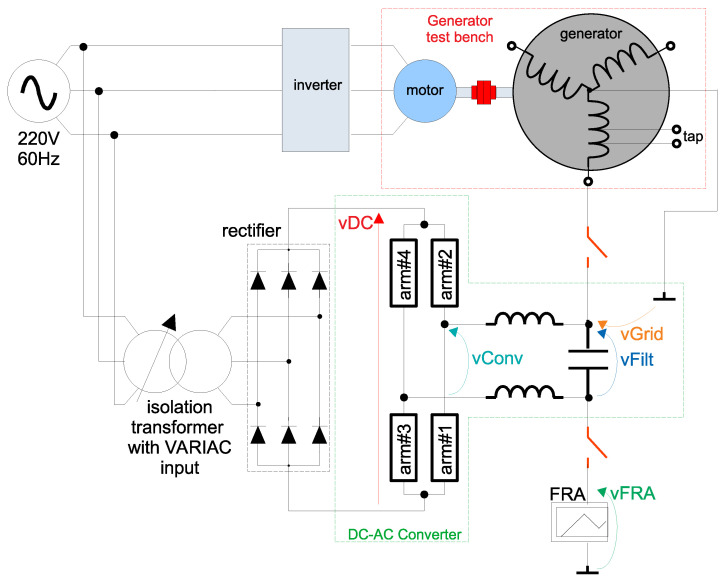
Schematics of the test setup.

In order to evaluate the idea of online FRA through active coupling, a synchronous generator was used as the machine under testing. Figure 8 presents the schematics of the test setup, where each part is explained as follows. The stator windings of this generator were specially constructed (as described in [39]) with taps, where parasitic elements can be inserted in order to simulate insulation failures. 

**Figure 9:**
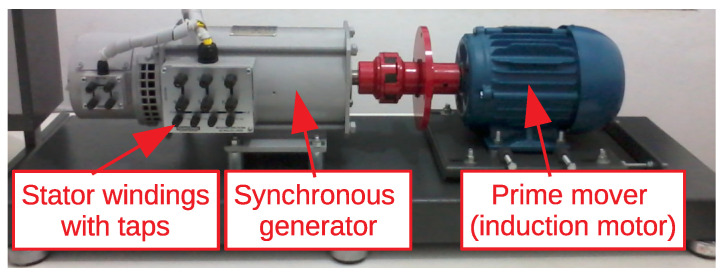
Test bench with 2 kW/220 V/60 Hz synchronous generator.

Figure 9 presents a photograph of the test bench with the 2 kW/220 V/60 Hz synchronous generator. An induction motor (fed by inverter) is used as the prime mover.

The FRA measurement circuit of Figure 1 was implemented on a STEMlab FPGA board, which has two high speed ADCs and two high speed DACs (both operating at a rate up to 125 Msps and 14 bits resolution). An example of implementation of the FRA firmware on the STEMlab board is presented in [40].

**Figure 10:**
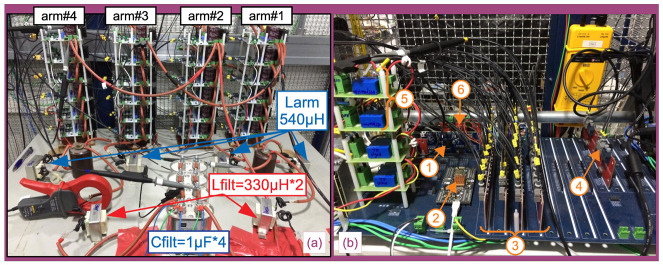
Test bench of the DC–AC converter: (**a**) power stage of the Modular Multilevel Converter (MMC) topology (bi-phase, 4 arms, N=6 SMs/arm); (**b**) control stage (gate signals sent to the power stage through optical fibers).

Figure 10 presents a photo of the bi-phase MMC DC–AC converter (with *N* = 6 SMs/arm), according to Figure 5. Subfigure (a) presents the power stage of the converter, whereas subfigure (b) presents the control stage. The DC input of the power stage (vDC) must be isolated from the grid voltage vGrid. In the case of the setup of Figure 8, the DC link is fed by a rectifier isolated from the main supply through a transformer—although any other source of isolated DC power can be used. Furthermore, in the particular case of the setup of Figure 8, a VARIAC is used at the primary side of the transformer, in order to be able to change the DC voltage to different values.

Each SM board receives their gate signals through optical fibers. A detailed project on these SM boards, including the gate drivers, is presented in [41].

The control loop of Section 2.3 was implemented in a Texas Instruments DSP (TMS320F28379D, on a ControlCard [42]). This DSP, however, has only 12 PWM channels. In order to control the 24 SMs of Figure 5 and Figure 10, a Xilinx FPGA (Zynq 7020, on a system-on-module snickerdoodle [43]) was used in order to implement the multilevel PWM. At each sampling time, the DSP performed the whole algorithm and sent (through SPI) the modulation indexes to the FPGA. The SPI communication between DSP and FPGA was performed physically in a mother board (presented in details in [41]). Figure 10b presents the mother board with the following accessory boards:

1. DSP TMS320F28379D ControlCard [42].
2. FPGA system-on-module snickerdoodle [43].
3. Four optical fiber interface boards between the FPGA and the SM boards. Each of these boards can interface to six SM boards and are presented in detail in [41].
4. Optical fiber interface between the DSP and a relay board (both boards presented in detail in [41]) that acted as the circuit breaker of Figure 3.
5. Hall effect sensors for vGrid and vConv of Figure 3. These boards isolated and converted the nominal voltages of the machine under testing and DC-AC converter to signals between $-10V_{peak}$ and $+10V_{peak}$, referenced to a common ground.
6. Analog signal conditioning board (presented in detail in [41]). These boards fit the output of the Hall effect sensors to the range of 0 V to $+3V_{peak}$ of the analog channels of the DSP.

### 3.1. Operation of the bi-Phase MMC Converter

**Figure 11:**
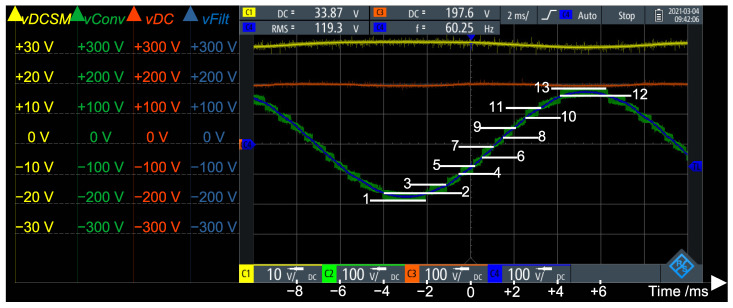
Open loop operation of the Modular Multilevel Converter (MMC) topology (bi-phase, 4 arms, N=6 SMs/arm ⇒13 levels).

Figure 11 presents the open loop operation of the MMC bi-phase converter, with the DC link voltage set to $v_{DC}=200$ V. During steady state operation, the total DC link voltage is divided by the *N* SMs in an arm. The plot in red is the total DC voltage of the converter $v_{DC}=200$ V. The yellow plot is the voltage at the DC capacitor of one of the SMs $v_{DCSM}=v_{DC}/N=200V/6=33$ V. The green plot is the output of the converter (non filtered) $v_{Conv}$. It can be noted that this voltage has 13 levels, as expected for *N* = 6 SMs/arm with modulation 2·N+1. The blue plot is the voltage after the LC filter $v_{Filt}$.

### 3.2. Active Voltage Blocking

**Figure 12:**
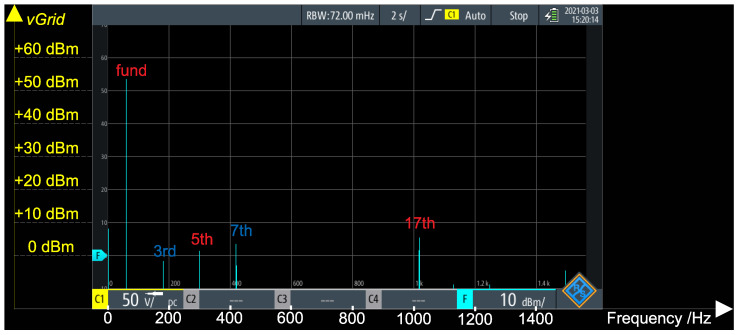
FFT of generator’s voltage $v_{Grid}$.

Figure 12 presents an FFT of the generator’s voltage ($v_{Grid}$ in Figure 3), under no load and without operation of the active voltage blocking. It can be noted that, besides the fundamental, there are components at the 3rd, 5th, 7th and 17th harmonics. In order to well cancel this voltage at the FRA point, the voltage control loop of the converter must track each of these components the best as possible.

With a sampling rate of $f_{s}=5$ kHz, the DSP should have no problems in tracking the fundamental (with 83 samples per 60 Hz cycle) and the 3rd (with 28 samples per 180 Hz cycle) harmonics. In case of the 5th (with 17 samples per 300 Hz cycle) and the 7th (with 12 samples per 420 Hz cycle) harmonics, a higher sampling frequency would improve the results. However, in order to well compensate the 17th harmonics (1020 Hz), the sampling frequency should be, at least, 20 kHz—which would imply in a shorter time frame in order to execute the whole algorithm and in a higher memory usage (in order to be able to store larger buffers, as the number of samples per cycle would increase dramatically). Hence, in the results that follow, there is no compensation for the 17th harmonics.

The results that follow have been obtained with the voltage control loop parameters defined in Table 2.

**Figure 13:**
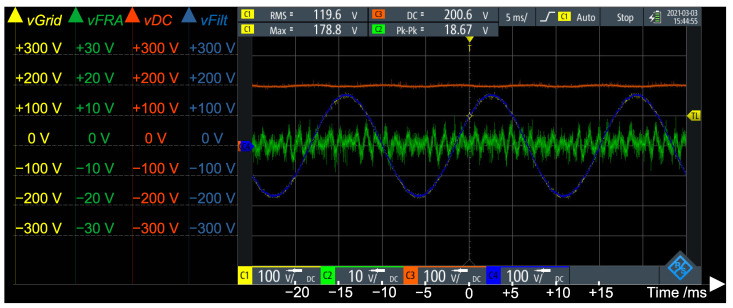
Operation of the active voltage blocker.

**Figure 14:**
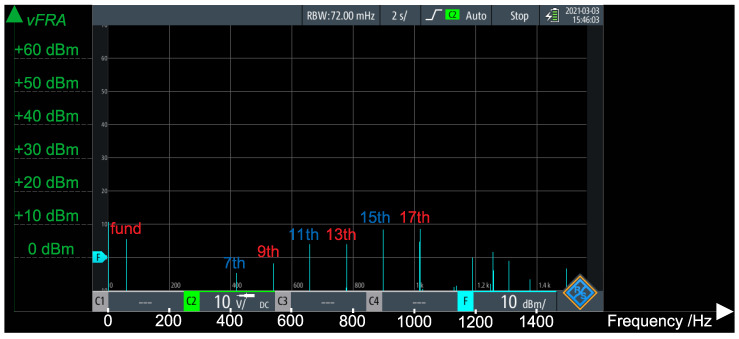
FFT of residue voltage $v_{FRA}$.

Initially, as shown in Figure 13 and Figure 14, the active voltage blocker was tested with a 1 kΩ resistor instead of the FRA analyzer of Figure 3. This resistor had the same value of the shunt resistor of the FRA measurement circuit of Figure 1.

Figure 13 presents the performance of the active voltage blocker. The red plot is the total DC link voltage of the converter, set at vDC=200 V. The yellow plot (which is superposed by the blue plot) is the generator’s voltage vGrid (around 220/$\sqrt{3}V_{RMS}$ = 180$V_{peak}$). The blue plot is the filtered voltage of the MMC converter, vFilt. It can be noted that it is, practically, superposed to vGrid, as expected. The green plot is the residual voltage at the 1 kΩ resistor. It can be noted that, although the blue and yellow plots are superposed (meaning that the converter was successfully tracking the generator’s voltage), there is still a residue. It is important to note that the scale of the green channel is magnified 10 times in relation to the others (10 V/division—in opposition to 100 V/division of the other channels).

Figure 14 presents the FFT of the residual voltage. When comparing this figure against Figure 12 (FFT of the generator’s voltage), it can be noted that the fundamental component has dramatically dropped and that the 3rd and 5th are eliminated. Due to the smaller number of samples per cycle, the 7th harmonics are still present—although attenuated to bellow 0 dBm (approximately 0.22$V_{RMS}$ = 0.31$V_{peak}$). However, it can be noted that the higher order harmonics (which are not compensated by the controller) have increased (although all of them are below 10dBm, approximately 0.707$V_{RMS}$ = 1$V_{peak}$).

This residual voltage, although not perfectly eliminated, allows the safe connection of the FRA equipment for online measurements, as will be presented in Section 3.4. First, Section 3.3 presents the offline measurements on the same machine, with the same fault progression—in order to well compare offline and online results.

### 3.3. Offline FRA

**Figure 15:**
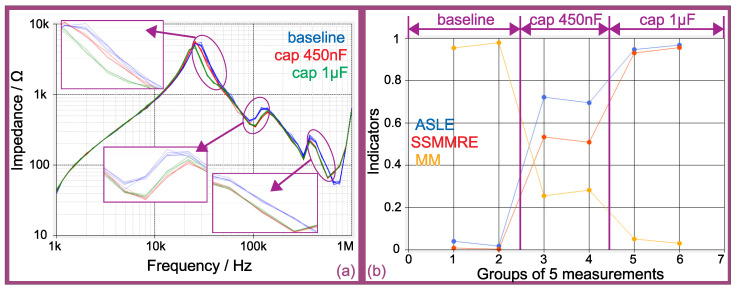
Offline FRA for a progression of turn insulation failure (capacitors inserted between taps): (**a**) impedance spectra; (**b**) trend curves of some statistical indicators.

Figure 15 presents the results of offline measurements on the synchronous generator of Figure 9. According to [15], besides temperature and humidity, the rotor position may have an influence on the measurements—hence, in order to achieve maximum repeatability in the measurements, all offline measurements have been performed with the rotor at a fixed position.

Figure 15a presents the offline impedance spectra for a progression of insulation failures (simulated with the insertion of parasitic capacitances between two taps representing 2% of the winding) on the generator’s stator. The choice of capacitances as the parasitic elements is based on the studies of Perisse et al. [44] and Madonna et al. [45], that show that winding capacitance increases with the insulation aging—hence, this is an easy way to simulate early damage on the insulation system. It is important to note that, although the parasitic capacitances inserted at the taps can only simulate insulation failures, the effect of any other type of failure (as long as they produce a change in the parameters of the windings) could be detected as well. In order to avoid visual pollution, the figure presents only five measurements at each insulation condition (although the complete set of measurements is used in order to calculate the trend curve of the statistical indicators):

- Blue for the baseline condition (three groups at baseline condition were measured, although only the first group is shown in the figure);
- Red for the condition with a capacitor of 450 nF inserted on the 2% tap (two groups at 450 nF condition were measured, although only the first group is shown in the figure);
- Green for the condition with a capacitor of 1 μF inserted on the 2% tap (two groups in 1 μF condition were measured, although only the first group is shown in the figure).

The first detail of Figure 15a (presenting a zoom on the region between 20 and 50 kHz) reveals a slight displacement between the five measurements at 450 nF from the five measurements at baseline. Furthermore, the five measurements at 1 μF are clearly displaced from the measurements at either 450 nF and baseline conditions.

The second and third details of Figure 15a (presenting zooms on the region between 80 kHz and 150 kHz and on the region between 400 and 600 kHz, respectively) show that both conditions with 450 nF and 1 μF are clearly displaced from the baseline—although they seem to be very close to each other.

It is important to note that interpretations of the displacements in relation to the baseline are not within the scope of this paper, as this would require an analysis of the equivalent circuit of the winding, and it would be far complex and distant from the goal of this paper (which is the proposition of the active coupling). Instead, apart from the physical interpretation of the displacements, we consider it enough to understand the displacements as variations of some of the parameters of the machine. The aging of the turn insulation of a generator was simulated with the insertion of the parasitic capacitors. However, some other displacements in the spectra could be visualized as well—as in the case of a mechanical deformation on the windings of a transformer, which also produces changes on the measured parameters.

In order to reduce the subjectivity concerning the displacements in relation to the baseline, as presented in Section 2.1, the literature proposes the use of statistical indexes (a survey on these indexes is presented in [17]) which quantify the displacements as numbers or scores. In order to ease the comparisons in case of noisy measurements (which is the case of online measurements), the average of the calculated statistical indexes among the same group of five measurements was plotted. This number of five measurements was chosen arbitrarily.

Each group of five measurements has been compared against the baseline condition (the average spectrum of the blue plots in Figure 15a) using the ASLE (Equation (2)), the SSMMRE (Equation (3)) and the MM (Equation (4)) indexes. It is important to note that each one of the indexes has a different scale of values. Thus, in order to ease the comparisons between them in a same figure, all of their scales have been normalized, considering their maximum values (max(idx)) and minimum values (min(idx)) according to Equation (11) [17]. Furthermore, some of the indexes, such as the ASLE and the SSMMRE, have higher values during worsening conditions (in relation to failure)—whereas some others, such as the MM, have lower values.

(11) $$idx_{norm}=\frac{idx-min(idx)}{max(idx)-min(idx)}$$

where idx is any one of the indexes.

Figure 15b presents the trend curve of the average of the selected statistical indexes. It can be noticed that the groups 1 and 2 have been identified as in similar condition to the baseline (and, indeed, these 3 groups of measurement were taken at the same condition). Furthermore, it can be noted the progression of failure, with groups 3 and 4 (measurements taken at 450 nF condition) at an intermediary stage and groups 5 and 6 (measurements taken at 1 μF condition) at a further level.

### 3.4. Online FRA

**Figure 16:**
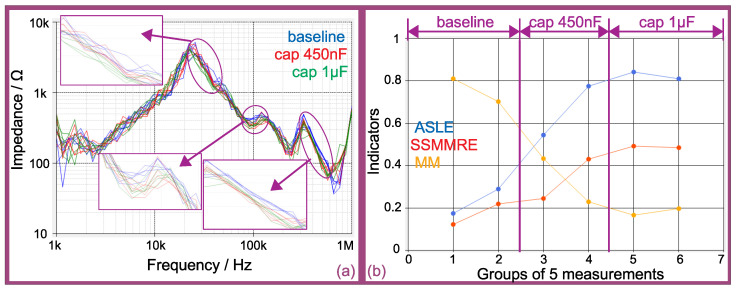
Online FRA for a progression of turn insulation failure (capacitors inserted between taps): (**a**) impedance spectra; (**b**) trend curves of some statistical indicators.

Figure 16 presents the results of online measurements on the synchronous generator of Figure 9 operating at 220$V_{RMS}$ phase-phase, with active coupling connected phase-ground (220/$\sqrt{3}V_{RMS}$ = 180$V_{peak}$).

Figure 16a presents the online impedance spectra for the exact same progression of failures of the offline case. Furthermore, groups of five measurements were taken under each condition.

The obvious conclusion after the comparison between Figure 15a and Figure 16a is that the online measurements are much noisier. One supposition for the noise is the influence of rotor position on the inductive regions of the spectrum (as studied in [15])—although, the electromagnetic noise (originated from either the inverter driving the prime mover and the power electronics converter of the active coupling) may have an important contribution on this matter. Apart from the noise, it is clear that the online measurements follow the same pattern of the offline ones.

Just like in the offline case, the zooms on the same regions of the spectra show the same conclusions (although, due to the noise, the measurements within a same group are more spread from each other): the zoom on the region between 20 and 50 kHz shows a slight displacement of the five measurements at 450 nF from the five measurements at baseline and a further displacement at 1 μF; the zooms on the other two regions show both 450 nF and 1 μF at the same displacement from the baseline.

As the online measurements are noisier than the offline ones, the average of the statistical indexes can give a much more objective result than the visual comparison. Figure 16b presents the trend curve of the average of the selected statistical indexes. It can be noted, just like in the offline case, that the groups 1 and 2 have been identified as in similar condition to the baseline (although they are more spread than in the offline case). Furthermore, it can be noted the progression of failure, with groups 3 and 4 (measurements taken at 450 nF condition) at an intermediary stage and groups 5 and 6 (measurements taken at 1 μF condition) at a further level.

It is important to note that the scope of this paper was the presentation of the concept of active coupling; hence, no advanced analysis was performed. Although, for example, the use of control charts [46] on the trend curves of the indicators would detect a variation outside the normal limits of statistical variations of the indicator, and would be appropriate for dealing with such noisier measurements.

## 4. Conclusions

This paper presented the development of an active coupling system for the online application of FRA to energized machines. The system is based on a power electronics converter and generates a voltage that cancels out the grid voltage at the terminals of the FRA equipment—hence, the FRA can be safely connected to the energized machine. Didactic descriptions of the principles of FRA and power electronics converters have been presented. The issue of passive coupling and the loss of low frequency regions on the spectra have been discussed. The proposed active coupling, on the other hand, is based on the cancellation of the grid voltage, allowing in theory the use of the whole spectrum.

A first implementation of the concept of the active coupling has been presented. This concept, however, is still experimental, with plenty of room for improvement. It can be noted that, although in perfect agreement with the offline results, the online results were clearly noisier than their offline counterparts. This noise could be related to the issue of variation of the inductive measurements in relation to the rotor position (as studied in [15]), as in the online case the rotor position of the generator is constantly varying—although a more realistic hypothesis is the contamination by electromagnetic noise (at the ADCs of the FRA equipment) generated by either the inverter of the prime mover of the test-bench or by the proposed power electronics converter itself. As the proposition was a proof-of-concept, no EMC test has been performed on the prototype—although it is an essential future step [47]. Apart from the noise at the measurements, the proposed concept has been proved.

It must be highlighted that the proposed system might also have applications in the power line communications/carrier system, as the issues of injecting a high frequency signal on an energized power line are the same.

## Figures and Tables

**Table 1:** PWM parameters for the bi-phase MMC with *N* = 6 SMs/phase of Figure 5.

arm#1 (${\mathrm{mod}}_{1}=0^{\circ }$)	arm#2 (${\mathrm{mod}}_{2}=180^{\circ }$)	arm#3 (${\mathrm{mod}}_{3}=180^{\circ }$)	arm#4 (${\mathrm{mod}}_{4}=0^{\circ }$)
$A_{l}\left(1\right)=0^{\circ }$	$A_{u}\left(1\right)=30^{\circ }$	$B_{l}\left(1\right)=0^{\circ }$	$B_{u}\left(1\right)=30^{\circ }$
$A_{l}\left(2\right)=60^{\circ }$	$A_{u}\left(2\right)=90^{\circ }$	$B_{l}\left(2\right)=60^{\circ }$	$B_{u}\left(2\right)=90^{\circ }$
$A_{l}\left(3\right)=120^{\circ }$	$A_{u}\left(3\right)=150^{\circ }$	$B_{l}\left(3\right)=120^{\circ }$	$B_{u}\left(3\right)=150^{\circ }$
$A_{l}\left(4\right)=180^{\circ }$	$A_{u}\left(4\right)=210^{\circ }$	$B_{l}\left(4\right)=180^{\circ }$	$B_{u}\left(4\right)=210^{\circ }$
$A_{l}\left(5\right)=240^{\circ }$	$A_{u}\left(5\right)=270^{\circ }$	$B_{l}\left(5\right)=240^{\circ }$	$B_{u}\left(5\right)=270^{\circ }$
$A_{l}\left(6\right)=300^{\circ }$	$A_{u}\left(6\right)=330^{\circ }$	$B_{l}\left(6\right)=300^{\circ }$	$B_{u}\left(6\right)=330^{\circ }$

**Table 2:** Parameters of the voltage controller.

Parameter	Value
$f_{s}$	5 kHz
$k_{p}$	0.60
$k_{r1}$	25.0
$\omega_{c1}$	π rad/s
$k_{r3}=k_{r5}$	15.0
$\omega_{c3}=\omega_{c5}$	2·π rad/s
$k_{r7}$	20.0
$\omega_{c7}$	2·π rad/s

## Data Availability

Not applicable.

## Abbreviations

The following abbreviations are used in this manuscript:

ADC	Analog-to-Digital Converter
CBM	Condition Based Maintenance
DAC	Digital-to-Analog Converter
DFT	Discrete Fourier Transform
DSP	Digital Signal Processor
FFT	Fast Fourier Transform
FPGA	Field Programmable Gate Array
FRA	Frequency Response Analysis
IFRA	Impulse Frequency Response Analysis
IGBT	Insulated-Gate Bipolar Transistor
MMC	Modular Multilevel Converter
PLC	Power-Line Communication/Carrier
PI	Proportional-Integral
PR	Proportional plus Resonant
PWM	Pulse Width Modulation
SFRA	Sweep Frequency Response Analysis
SM	Submodule
SPI	Serial Peripheral Interface
THD	Total Harmonic Distortion

## Appendix A. Amplitude Extraction through DFT

Given a discretized signal *v* (which in Equation (1) could be either the signal ${\vec{V}}_{2}$ or the resulting subtraction of ${\vec{V}}_{1}$ and ${\vec{V}}_{2}$), composed of $N_{s}$ samples, obtained at a $f_{sFRA}$ sampling rate (not to be confused with the sampling rate of the voltage controller loop, $f_{s}$), the amplitude of the component at the frequency $f_{HF}$ (i.e., the high frequency being injected in the machine) can be extracted using the well known DFT procedure [48]. This procedure is based on the correlation between the signal *v* and a cosinusoidal wave at frequency $f_{HF}$, which results its real component (calculated as Equation (A1)), and on its correlation with a sinusoidal wave (also at frequency $f_{HF}$), which results in its imaginary component (calculated as Equation (A2)).

(A1) $$Re_{vHF}=\sum_{k=0}^{N_{s}-1}v\left(k\right)\cdot \cos\left(2\cdot \pi \cdot \frac{f_{HF}}{f_{sFRA}}\cdot k\right).$$

(A2) $$Im_{vHF}=\sum_{k=0}^{N_{s}-1}v\left(k\right)\cdot \sin\left(2\cdot \pi \cdot \frac{f_{HF}}{f_{sFRA}}\cdot k\right).$$

The amplitude of the component at frequency $f_{HF}$ is, then, calculated as the absolute value of the real and imaginary components as equation (A3).

(A3) $$\left|v_{HF}\right|=\frac{\sqrt{Re_{vHF}^{2}+Im_{vHF}^{2}}}{N_{s}/2}.$$
